# „Skills fOr Life Adjustment and Resilience“ Programm: Kurzintervention zur Reduktion anhaltender subklinischer Belastung nach Katastrophen und anderen schweren Belastungen

**DOI:** 10.1007/s00278-021-00535-0

**Published:** 2021-09-15

**Authors:** Annett Lotzin, Imke Hinrichsen, Laura Kenntemich, Renée-Christin Freyberg, Winnie Lau, Kari Gibson, Meaghan O’Donnell

**Affiliations:** 1 Klinik für Psychiatrie und Psychotherapie, Universitätsklinikum Hamburg-Eppendorf, Hamburg, Deutschland; 2 Phoenix Centre for Posttraumatic Mental Health, The University of Melbourne, Melbourne, Australien; 3 Martinistr. 52, 20246 Hamburg, Deutschland

**Keywords:** Katastrophen, Trauma, Kurzinterventionen, Subsyndromal, Sekundärprävention, Disasters, Trauma, Brief interventions, Subsyndromal, Secondary prevention

## Abstract

**Hintergrund:**

Nach Katastrophen, Traumata und anderen schweren Belastungen entwickelt ein Teil der Betroffenen eine psychische Erkrankung, während ein weiterer Teil anhaltende subklinische Belastungen zeigt, die das psychosoziale Funktionsniveau einschränken. Anhaltend subklinisch belastete Menschen erhalten jedoch selten psychosoziale Unterstützungsangebote.

**Ziel der Arbeit:**

Vorgestellt wird das Programm „Skills fOr Life Adjustment and Resilience“ (SOLAR), eine Kurzintervention für Menschen mit anhaltender subklinischer Belastung nach Katastrophen und anderen schweren Belastungen. Erste Ergebnisse einer Pilotstudie zur Zufriedenheit mit dem Programm bei Betroffenen werden berichtet.

**Methoden:**

Das SOLAR-Programm wurde von einem internationalen Konsortium, zusammengesetzt aus Katastrophen- und Traumaexperten, entwickelt. Es beinhaltet 5 wöchentliche Gruppensitzungen, in denen verhaltenstherapeutische Elemente vermittelt werden. Im Rahmen der Pilotstudie nahmen 15 Teilnehmende im Präsenzformat und 15 Teilnehmende mithilfe einer Videokonferenzschaltung während der „Coronavirus Disease 2019“ (COVID-19) Pandemie am SOLAR Programm teil. Nach Abschluss beantworteten die Teilnehmenden den Fragebogen zur Messung der Patientenzufriedenheit (ZUF-8).

**Ergebnisse:**

Die Teilnehmenden waren „weitgehend“ bis „sehr zufrieden“. In der Präsenzgruppe war die Zufriedenheit über alle Aspekte hinweg geringfügig stärker ausgeprägt als in der Onlinegruppe. Die TrainerInnen bewerteten das Programm als gut durchführbar.

**Schlussfolgerung:**

Das SOLAR-Programm ist eine vielversprechende Kurzintervention bei anhaltender subklinischer Belastung nach schweren Belastungen, die im Präsenz- oder im videogestützten Format weiter auf seine Wirksamkeit erprobt werden sollte. Im Beitrag werden Praxisempfehlungen zur Durchführung gegeben.

## Abstract (en)

**Background:**

After catastrophes, trauma and other highly stressful experiences, a proportion of individuals develop mental illness, while another proportion exhibit persisting subclinical distress that limits psychosocial functioning. Individuals with persisting subclinical distress rarely receive psychosocial support services.

**Aim of the study:**

This paper presents the Skills fOr Life Adjustment and Resilience (SOLAR) program, a brief intervention developed for people with persistent subclinical distress following catastrophes and other severe stressors. In addition, we report the preliminary findings of a pilot study on the satisfaction with the program in face-to-face and video conferencing formats among people affected by traumatic experiences.

**Methods:**

An international consortium of catastrophe and trauma experts developed the SOLAR program. It includes five weekly group sessions in which behavioral therapy elements are taught. In a pilot study 15 participants received the SOLAR group program in a face-to-face format, and 15 received the program via video conferencing during the first weeks of the Coronavirus Disease 2019 (COVID-19) pandemic. After program completion, we assessed satisfaction with the program using the German version of the client satisfaction questionnaire (CSQ-8).

**Results:**

Participants were “largely” to “very satisfied” with the SOLAR program. The satisfaction in the face-to-face group was slightly greater in all aspects than in the online group. The trainers rated the program as easy to implement.

**Conclusion:**

The SOLAR program represents a promising brief intervention for persistent subclinical distress after severe stressors, which should be further tested for its effectiveness in face-to-face or video conferencing formats. Practical recommendations for implementation are provided.

Nach Katastrophen, potenziell traumatischen Erfahrungen oder anderen schweren Belastungen erhalten Betroffene mit anhaltender subklinischer Belastung oftmals erst Monate oder Jahre später psychosoziale Unterstützung. Unter den Gesichtspunkten des persönlichen Leids und der Kosteneffektivität erscheint es jedoch sinnvoll, den Betroffenen frühzeitig Kurzinterventionen zugänglich zu machen, bevor sich Vollbilder psychischer Störungen entwickeln. Von einem internationalen Konsortium, zusammengesetzt aus Katastrophen- und Traumaexperten, wurde hierzu das einfach anzuwendende „Skills fOr Life Adjustment and Resilience (SOLAR)“ Programm entwickelt. Dieses Kurzprogramm niedriger Intensität kann innerhalb eines gestuften Versorgungsmodells eingesetzt werden.

## Hintergrund und Fragestellung

Als Folge des Klimawandels und internationaler Konflikte nehmen Naturkatastrophen (z. B. Überschwemmungen, Buschfeuer) und menschliche Katastrophen (z. B. Massengewalt, Terrorismus) weltweit zu. Nach solchen Ereignissen haben Betroffene ein erhöhtes Risiko, anhaltend belastet zu sein und eine psychische Erkrankung, wie z. B. eine posttraumatische Belastungsstörung (PTBS; Beaglehole et al. 2018), eine Angststörung (Fergusson et al. 2014) oder eine depressive Störung (Beaglehole et al. 2018) zu entwickeln. Somit verursachen Katastrophen enorme gesundheitliche, wirtschaftliche und soziale Kosten (North und Pfefferbaum 2013) und werden von globalen Gesundheitsorganisationen als eines der vordringlichsten Probleme der öffentlichen Gesundheit unserer Zeit betrachtet (United Nations Office for the Coordination & of Humanitarian Affairs, 2016).

Nach Katastrophen, potenziell traumatischen Erfahrungen oder anderen schweren Belastungen erhalten Betroffene mit anhaltender subklinischer Belastung oftmals erst nach Monaten oder Jahren psychosoziale Unterstützung (Korte et al. 2016). Da sie die Kriterien für eine psychische Störung nicht erfüllen, werden sie von psychotherapeutischen Behandlungen ausgeschlossen (Korte et al. 2016). Betroffene werden oftmals erst dann (psychotherapeutisch) behandelt, wenn sich eine psychische Störung entwickelt hat (Forbes et al. 2010). Unter den Gesichtspunkten des persönlichen Leids und der Kosteneffektivität erscheint es jedoch sinnvoll, Betroffenen mit anhaltender subklinischer Belastung frühzeitig Kurzinterventionen zugänglich zu machen, bevor sich Vollbilder psychischer Störungen entwickelt haben (O’Donnell et al. 2016).

**Figure 1:**
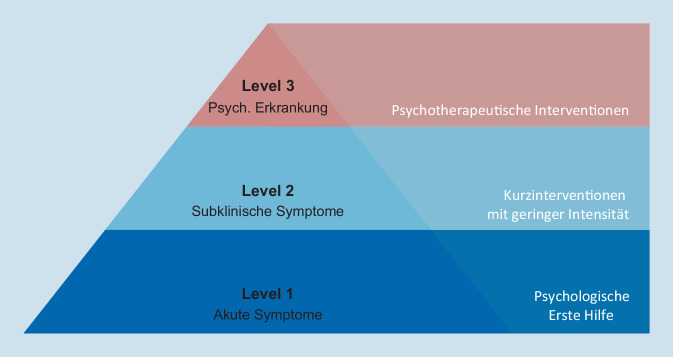

Um eine angemessene Behandlung von Betroffenen nach Katastrophen und anderen Traumata zu gewährleisten, wurden gestufte Versorgungsansätze vorgeschlagen (Abb. 1; NATO Joint Medical Committee 2008). Ein solches Versorgungsmodell beinhaltet ein systematisches Screening der Symptombelastung in der Frühphase nach einer Katastrophe oder einer anderen schweren Belastung, und falls notwendig, die Zuweisung zu einer Intervention auf einem angemessenen Intensitätsniveau (Zatzick et al. 2013). Bei anhaltender subklinischer Belastung oder bei Vorliegen einer Anpassungsstörung (d. h. bei Symptomen wie Grübeln über das belastende Ereignis, Schlafstörungen und ein beeinträchtigtes psychosoziales Funktionsniveau) erhalten Betroffene Kurzinterventionen, bei denen die Förderung der Erholung von der Belastung im Vordergrund steht. Falls Betroffene trotz Teilnahme an diesen Maßnahmen weiterhin anhaltend belastet sind, erhalten sie in der nächsten Stufe intensivere psychotherapeutische Behandlungsangebote. Es gibt Hinweise darauf, dass gestufte Versorgungsmodelle im Vergleich zur (nichtgestuften) Standardversorgung die Entwicklung psychischer Störungen effektiver verhindern können und kosteneffizienter sind (Cohen et al. 2017). Aktuell sind gestufte Versorgungsmodelle in Deutschland jedoch nicht die gängige Praxis.

Bislang liegen kaum Kurzinterventionen für Menschen mit anhaltender subklinischer Belastung vor. Die am besten erforschte Kurzintervention, die auf leichte bis mittlere Belastungssymptome abzielt, ist das Problem Management Plus (PM+; Dawson et al. 2015). Das Programm adressiert störungsübergreifende Symptome anhaltender psychischer Belastung sowie leichte depressive oder Angstsymptome (Dawson et al. 2015). Das PM+ umfasst evidenzbasierte Strategien für Stress- und Problemmanagement, Verhaltensaktivierung und soziale Unterstützung und wurde speziell für den Einsatz in Entwicklungsländern entwickelt (Dawson et al. 2015). Es zeigte sich in 2 Studien in Pakistan (Rahman et al. 2016) und bei von Gewalt betroffenen Frauen in Kenia (Bryant et al. 2017) als effektiv, psychische Belastung zu senken.

„Skills for Psychological Recovery“ (SPR; National Centre for PTSD and National Child Traumatic Stress Network 2009) ist eine Kurzintervention, die speziell dafür entwickelt wurde, Belastungen nach Katastrophen zu senken. In 6 Sitzungen werden Fertigkeiten wie Problemlösefähigkeiten, die Förderung positiver Aktivitäten, die Förderung hilfreicher Gedanken und der Aufbau sozialer Beziehungen vermittelt (Wade et al. 2014). Obwohl das SPR-Programm speziell für Überlebende von Katastrophen entwickelt worden ist, beinhaltet es keine Intervention, die eine Verarbeitung traumabezogener Erinnerungen ermöglicht (Cusack et al. 2016). Studienbefunde zur Effektivität wurden bislang nicht veröffentlicht.

Vor dem Hintergrund dieser Forschungs- und Versorgungslücke einer einfach anzuwendenden Kurzintervention zur Reduktion anhaltender subklinischer Belastung nach Katastrophen und anderen schweren Belastungen wurde 2016 vom Phoenix Australia Centre for Posttraumatic Mental Health der Universität Melbourne (Forbes et al. 2017) ein internationales Konsortium aus Katastrophen- und Traumaexperten ins Leben gerufen, um eine neue Intervention zu entwickeln: das SOLAR-Programm. Die Expertengruppe entwickelte dieses Kurzprogramm niedriger Intensität für von Katastrophen und Traumata Betroffene; es kann im Rahmen eines gestuften Versorgungsmodells eingesetzt werden (O’Donnell et al. 2020).

Das SOLAR-Programm basiert auf verhaltenstherapeutischen Strategien, die sich in psychotherapeutischen Interventionen als effektiv erwiesen haben. Diese umfassen Verhaltensaktivierung, Aufrechterhaltung eines gesunden Lebensstils, Verbesserung der sozialen Unterstützung, Emotions- und Stressregulierung sowie kognitiv-behaviorale Strategien zur Kontrolle von Grübelgedanken. Da das Programm speziell für Überlebende von Katastrophen und anderen potenziellen Traumata entwickelt wurde, beinhaltet es ein traumafokussiertes Interventionselement, das auf die emotionale Verarbeitung des traumatischen Ereignisses abzielt (O’Donnell et al. 2020). Das Programm ist leicht zu erlernen und kann auch von geschulten psychosozialen Gesundheits- oder Gemeindefachkräften durchgeführt werden, was seine potenzielle Reichweite nach Großschadensereignissen, Katastrophen und anderen sehr belastenden Erfahrungen erhöht. Sollte sich das SOLAR-Programm als effektiv erweisen, psychische Belastung zu senken, könnte die Intervention ein hohes Potenzial haben, eine Lücke in der Gesundheitsversorgung im Rahmen eines gestuften Versorgungsmodells nach Katastrophen und anderen schweren Belastungen zu schließen.

Das SOLAR-Programm wurde zunächst bei Überlebenden australischer Buschbrände erprobt (O’Donnell et al. 2020). Diese erste Pilotstudie liefert erste Anhaltspunkte dafür, dass die Intervention von geschulten Fachkräften sicher durchgeführt werden kann und von den Teilnehmenden gut angenommen wird (O’Donnell et al. 2020). Weitere Studien sind jedoch erforderlich, um die Durchführbarkeit, Akzeptanz und Wirksamkeit des SOLAR-Programms in verschiedenen Belastungskontexten zu untersuchen.

In diesem Beitrag werden Ergebnisse einer ersten deutschen Pilotstudie berichtet, bei der anhaltend subklinisch belastete Teilnehmende mit verschiedenen traumatischen Erfahrungen am SOLAR-Gruppenprogramm teilnahmen. Hierbei wurde ihre Zufriedenheit mit dem Programm im Präsenz- und erstmals auch in einem Onlineformat erhoben. Letzteres wurde während der durch die „Coronavirus Disease 2019“ (COVID-19) ausgelösten Pandemie videokonferenzgestützt durchgeführt. Darüber hinaus werden im Folgenden Praxisempfehlungen zur Durchführung des Programms gegeben.

## Methoden

### Beschreibung des SOLAR-Programms

**Figure 2:**
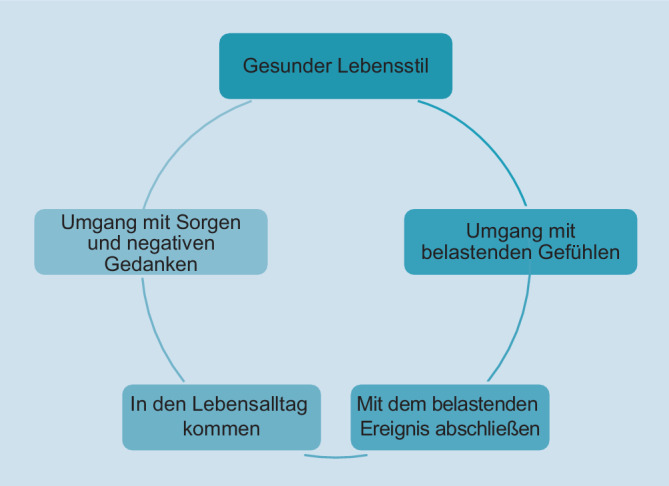

Das SOLAR-Programm wurde als psychosoziale Kurzintervention entwickelt, die auf verhaltenstherapeutischen Elementen basiert. Das Programm zielt darauf ab, subklinische psychische Belastung und Anpassungsstörungssymptome, wie z. B. Grübeln, Schlafstörungen und eingeschränkte psychosoziale Funktionen nach Katastrophen und anderen schweren Belastungen zu reduzieren. Das Programm umfasst 5 wöchentliche Sitzungen, in denen 6 Module enthalten sind (Abb. 2): 1. Gesunder Lebensstil; 2. Umgang mit belastenden Gefühlen; 3. Ins Leben zurückfinden; 4. Einen Abschluss mit dem belastenden Ereignis finden; 5. Umgang mit Sorgen und Grübeln sowie 6. Aufrechterhaltung gesunder Beziehungen. Im ersten Modul („Gesunder Lebensstil“) werden die Bedeutung körperlicher Aktivitäten und guter Schlafgewohnheiten für das allgemeine Wohlbefinden besprochen. Im zweiten Modul („Umgang mit belastenden Gefühlen“) werden Achtsamkeitsübungen eingeführt, die einen besseren Umgang mit belastenden Gefühlen fördern sollen. Im dritten Modul („Ins Leben zurückfinden“) vermitteln die TrainerInnen Fertigkeiten zum Aktivitätenaufbau. Hierbei werden Fertigkeiten zur Planung und zur Durchführung von wichtigen, angenehmen und bislang vermiedenen Aktivitäten erlernt. Neben dem Erlernen von Fertigkeiten beinhaltet das SOLAR-Programm im vierten Modul („Einen Abschluss mit dem belastenden Ereignis finden“) eine Schreibaufgabe, bei der die emotionale Verarbeitung des potenziell traumatischen Ereignisses im Vordergrund steht. Hierzu werden die Teilnehmenden gebeten, das traumatische oder sehr belastende Erlebnis in Form einer Geschichte aufzuschreiben. Die Erfahrungen mit der Schreibaufgabe werden in der sich anschließenden Sitzung gemeinsam besprochen, ohne jedoch auf die Inhalte der potenziell traumatischen Erfahrung einzugehen. Das Erlernen von Strategien zum Umgang mit negativen Gedanken und Grübeln wird im 5. Modul thematisiert („Umgang mit Sorgen und Grübeln“). Im letzten Modul („Aufrechterhaltung gesunder Beziehungen“) wird die Bedeutung zwischenmenschlicher Beziehungen vermittelt. Es werden Strategien erlernt, um förderliche soziale Kontakte zu pflegen. Eine ausführlichere Beschreibung der Programminhalte findet sich in englischer Sprache bei O’Donnell et al. (2020).

Die Teilnehmenden erhalten ein Arbeitsbuch, das die Sitzungsinhalte umfasst, sowie Arbeitsblätter zu den Übungen im Alltag und eine Dokumentation des Fortschritts. Jede Sitzung beinhaltet das Erlernen neuer Fertigkeiten und die konkrete Planung der Anwendung dieser Fertigkeiten zwischen den Sitzungen im Alltag. Das Programm kann im Einzel- und auch im Gruppensetting angewendet werden. Es ist darauf ausgelegt, von TrainerInnen in wöchentlichen Sitzungen angewendet zu werden. Personen, die dieses Programm durchführen, müssen keinen psychiatrischen oder psychologischen beruflichen Hintergrund haben – vielmehr wurde SOLAR für Laien entwickelt, wie Krankenpfleger, Freiwillige oder Notfallhelfer. Die TrainerInnen werden vorab von einer/einem „SOLAR Train the TrainerIn“ geschult, um das Programm nach einem standardisierten Manual durchzuführen. Bei der „SOLAR Train the TrainerIn“ handelt es sich um eine psychotherapeutisch geschulte Fachkraft, die Erfahrungen mit der Durchführung des SOLAR-Programms aufweist. Der/die SOLAR Train the TrainerIn supervidiert wöchentlich die TrainerInnen während der Durchführung des Programms.

### Studiendesign

Die berichteten sekundären Daten zur Behandlungszufriedenheit wurden in einer randomisierten kontrollierten Machbarkeitsstudie des SOLAR-Programms erhoben (Lotzin et al. in press). In der Machbarkeitsstudie wurden Prä- und Postdaten bei den Teilnehmenden erhoben. Im Rahmen der Sekundäranalyse wurden Daten zur Zufriedenheit mit der Intervention nach Abschluss des SOLAR-Programms ausgewertet. Alle Teilnehmenden wurden mithilfe einer Onlineumfrage über das Portal LimeSurvey befragt. Hierfür erhielten sie nach Abschluss des Programms eine E‑Mail mit dem Link zur Befragung. Die Datensätze der Teilnehmenden wurden in pseudonymisierter Form gespeichert.

### Stichprobe

Es wurden 30 Teilnehmende in die Studie eingeschlossen; diese erfüllten folgende Einschlusskriterien: (1) mindestens 18 Jahre alt; (2) Exposition mit einem potenziell traumatischen Ereignis gemäß dem PTBS-A-Kriterium der 5. Auflage des Diagnostic and Statistical Manual of Mental Disorders (DSM-5) und (3) leichte bis moderate subklinische Symptome von Angst, Depression oder PTBS (Testwertebereich der Generalized Anxiety Disorder 7 von 5–15; Patient Health Questionnaire‑9 von 5–15 oder PTSD Checklist for DSM‑5 von 5–32) oder eine Beeinträchtigung der sozialen, beruflichen oder alltäglichen Funktionsfähigkeit (Wertebereich der Functional Impairment Scale des Patient Health Questionnaire‑9 von 1–3). Ausschlusskriterium war (1) die Diagnose einer aktuellen psychischen Erkrankung mit Ausnahme der Anpassungsstörung (International Neuropsychiatric Interview Plus 7, M.I.N.I.; Sheehan et al., 1998). Eine eingehendere Beschreibung der Messinstrumente zur Prüfung der Ein- und Ausschlusskriterien findet sich bei Lotzin et al. (2021; eingereicht).

### Messinstrumente

#### Traumatisches Ereignis

Die Exposition mit einem Trauma wurde gemäß den PTBS-A-Kriterien des DSM‑5 für (American Psychiatric Association 2013) erfasst. Die Teilnehmenden wurden gefragt, ob sie mit tatsächlichem oder drohendem Tod, tatsächlichen oder drohenden schweren Verletzungen oder tatsächlicher oder drohender sexueller Gewalt konfrontiert waren. Zusätzlich mussten die Teilnehmenden für jede Art von Trauma angeben, ob sie es (a) selbst erlebt, (b) miterlebt oder (c) von anderen erfahren haben (z. B. erfahren, dass ein naher Verwandter oder enger Freund einem Trauma ausgesetzt war). Zusätzlich wurde erfragt, wie lange das traumatische Ereignis zurückliegt.

#### Zufriedenheit mit dem Programm

Zur Erfassung der Zufriedenheit mit dem SOLAR-Programm wurde der Fragebogen zur Messung der Patientenzufriedenheit (ZUF‑8; Schmidt et al. 1989) eingesetzt. Der ZUF‑8 stellt die deutsche Adaptation (Schmidt et al. 1989) des weit verbreiteten Client Satisfaction Questionnaire (CSQ‑8; Larsen et al. 1979) dar. Das Instrument erfasst die Behandlungszufriedenheit auf 4‑stufigen Bewertungsskalen (z. B. 1: schlecht bis 4: ausgezeichnet) anhand von 8 Items. Dabei werden 8 verschiedene Aspekte der Zufriedenheit erfasst, wie z. B. die eingeschätzte Qualität der Behandlung (Tab. 2). Der ZUF‑8 ist als unidimensionales Instrument konzipiert, das einen Schätzwert für die allgemeine Behandlungszufriedenheit liefert. Entsprechend kann ein Gesamtsummenwert berechnet werden, der von 8 bis 32 reicht; höhere Werte geben eine größere Zufriedenheit an. Für den ZUF‑8 liegen Hinweise auf seine gute Reliabilität (Cronbachs α = 0,87–0,93; Schmidt et al. 1989) und konvergente Validität vor (Körner et al. 2017).

### Studienablauf

Die Studie wurde von der Lokalen Psychologischen Ethikkommission am Zentrum für Psychosoziale Medizin (LPEK) am Universitätsklinikum Hamburg-Eppendorf genehmigt (LPEK-0068). Beworben wurde die Studie über das Internet (z. B. Ebay-Anzeigen, Facebook, Freizeitinteressengruppen) sowie über Flyer in Supermärkten, Apotheken, Cafés, Sportvereinen und Zeitungsanzeigen. Studieninteressenten wurden telefonisch kontaktiert und über die Studienziele und den Studienablauf sowie über die Inhalte des SOLAR-Gruppenprogramms aufgeklärt. Interessenten füllten zur Prüfung der Ein- und Ausschlusskriterien der Studie einen Onlinefragebogen aus; im Anschluss wurde von einer geschulten Psychologiestudentin ein strukturiertes klinisches Interview zur Diagnosestellung psychischer Erkrankungen durchgeführt. Wenn alle Studienkriterien erfüllt waren und die Person einwilligte teilzunehmen, wurde sie in die Studie eingeschlossen. Das SOLAR-Programm wurde in Gruppen von 5 bis 9 Teilnehmenden von jeweils einer TrainerIn und einer KotrainerIn durchgeführt. Als TrainerInnen wurden diejenigen Personen ausgewählt, die bereits Vorerfahrungen mit dem SOLAR-Programm hatten. Die TrainerIn leitete die Sitzungen überwiegend; die KotrainerIn unterstützte die TrainerIn administrativ, bei der Anleitung von Entspannungs- oder Achtsamkeitsübungen und bei der Besprechung der Hausaufgaben.

Das Programm umfasste eine 100-minütige Einführungssitzung und 4 weitere 90-minütige Sitzungen, die einmal wöchentlich stattfanden. Die Hälfte der Teilnehmenden (*n* = 15) erhielt das Programm im Präsenzformat, die verbleibende Hälfte (*n* = 15) erhielt das Programm onlinebasiert mithilfe einer Videokonferenzplattform. Das Programm wurde onlinebasiert in gleicher Weise durchgeführt wie in der präsenzbasierten Version. Die TrainerInnen setzten sich aus einer sozialpsychiatrischen Gesundheitsfachkraft mit kunsttherapeutischem Hintergrund, einer psychologischen Psychotherapeutin und 3 klinischen Psychologiestudierenden zusammen. Vorab wurden die TrainerInnen und KotrainerInnen von einer SOLAR Train the TrainerIn geschult. Bei der SOLAR Train the TrainerIn handelte es sich um eine psychologische Psychotherapeutin mit Expertise im Bereich der Behandlung von Traumafolgestörungen (Erstautorin), die zuvor von einer der Autorinnen des SOLAR-Programms (Meaghan O’Donnell) ausgebildet worden war. Die SOLAR Train the TrainerIn supervidierte die TrainerInnen und KotrainerInnen während der Durchführung des 5‑wöchigen Programms. Die Teilnehmenden bewerteten nach Abschluss des Programms ihre Zufriedenheit anhand eines Onlinefragebogens. Die erhobenen Daten wurden in pseudonymisierter Form gespeichert und ausgewertet. Die Befragung fand zwischen Dezember 2019 und März 2020 statt.

### Ableitung von Praxisempfehlungen zur Durchführung des SOLAR-Programms

Auf der Grundlage der gemachten Erfahrungen in der Durchführung des SOLAR-Programms entwickelten die durchführenden TrainerInnen gemeinsam Empfehlungen zur Durchführung. Hierzu dokumentierten die TrainerInnen in schriftlicher Form Aspekte, die sie für die Durchführung der Intervention als bedeutsam einschätzten. Die Aufzeichnungen der TrainerInnen wurden im Anschluss zusammengefasst, gemeinsam diskutiert und konsentiert.

## Ergebnisse

### Stichprobe

Es wurden 30 Teilnehmende randomisiert der Präsenz- oder Onlinegruppe zugeteilt. Insgesamt brachen 6 (20,0 %) der 30 randomisierten Teilnehmenden die Studie ab. Von den 15 randomisierten Teilnehmern der Präsenzgruppe brachen 2 (13,3 %) Teilnehmende die Studie ab: ein Teilnehmender vor Beginn des SOLAR-Programms, ein weiterer Teilnehmender nach der ersten Sitzung. Von den 15 randomisierten Teilnehmenden der Onlinegruppe brachen 4 Teilnehmende (26,7 %) die Studie ab: Drei der Teilnehmenden nach Abschluss der Datenerhebung vor Beginn des SOLAR-Programms und ein Teilnehmender nach der dritten Sitzung. Die Teilnehmenden waren zwischen 22 und 63 Jahre alt; das durchschnittliche Alter betrug 41 Jahre (Tab. 1). Mehr als die Hälfte war weiblich. Ewa ein Fünftel der Stichprobe berichtete ein sehr geringes monatliches Haushaltseinkommen von weniger als 1000 €.

**Table 1:** 

Variable	Präsenzgruppe (*n* = 15)		Onlinegruppe (*n* = 15)	
	*n*	%	*n*	%
*Geschlecht (männlich)*	*11*	*73,3*	*8*	*53,3*
*Familienstand*				
Unverheiratet	8	53,3	8	53,3
Verheiratet	5	33,3	5	33,3
Geschieden	2	13,3	2	13,3
*Partnerschaft*				
Ledig	7	46,7	5	33,3
Beziehung, zusammenlebend	5	33,3	7	46,7
Beziehung, getrennt lebend	3	20,0	3	20,0
*Kinder (ja)*	*7*	*46,7*	*5*	*33,3*
*Wohnsituation*				
Allein	4	26,7	8	53,3
Mit Partner/Kindern	7	46,7	4	26,7
Mit Freunden	2	13,3	0	0,0
Mit anderen	2	13,3	0	0,0
*Beruflicher Status*				
Nicht berufstätig	7	46,7	8	53,3
Teilzeit	2	13,3	3	20,0
Vollzeit	6	40,0	4	26,7
*Berufsgruppe*				
Angestellt	7	46,7	4	26,7
Selbstständig/Freiberufler	1	6,7	3	20,0
Schüler/Student	3	20,0	5	33,3
Hausfrau/Hausmann	0	0,0	1	6,7
Arbeitslos	3	20,0	0	0,0
(Früh- oder regulär) im Ruhestand	1	6,7	2	13,3
*Haushaltseinkommen (Netto)*				
Weniger als 1000 €	1	6,7	5	33,3
1000 bis weniger als 2000 €	7	46,7	0	0
2000 bis weniger als 3000 €	2	13,3	6	40,0
3000 bis weniger als 5000 €	3	20,0	2	13,3
Mehr als 5000 €	1	6,7	2	13,3
Möchte ich nicht berichten	1	6,7	0	0,0

*SOLAR* „Skills for Life Adjustment and Resilience Program“

Alle Teilnehmenden berichteten mindestens ein traumatisches Ereignis. Die am häufigsten berichteten Formen umfassten das Erleben einer lebensbedrohlichen Erkrankung oder Verletzung, einen sexuellen Übergriff oder eine andere ungewollte sexuelle Handlung oder den plötzlichen gewaltsamen Tod eines geliebten Menschen (Präsenzgruppe: lebensbedrohliche Erkrankung oder Verletzung [*n* = 6], sexueller Übergriff [*n* = 2], andere unerwünschte sexuelle Handlung [*n* = 3], gewalttätiger Angriff [*n* = 1], Gefangenschaft [*n* = 1], schweres menschliches Leid [*n* = 1], plötzlicher gewalttätiger Tod [*n* = 1]; Onlinegruppe: lebensbedrohliche Erkrankung oder Verletzung [*n* = 3], sexueller Übergriff [*n* = 3], schweres menschliches Leid [*n* = 1], plötzlicher gewalttätiger Tod [*n* = 3], Feuer oder Explosion [*n* = 1], schwerer Unfall [*n* = 1], Angriff mit einer Waffe [*n* = 2], plötzlicher Unfalltod [*n* = 1]). In der Präsenzgruppe lag das traumatische Ereignis im Durchschnitt 7,42 Jahre zurück (Standardabweichung [SD] ± 8,26 Jahre; Median [Md] = 4,00 Jahre; Min = 0,08 Jahre; Max = 24,00 Jahre). In der Online-Gruppe waren dies im Durchschnitt 10,34 Jahre (SD ± 13,19 Jahre; Md = 4,00 Jahre; Min = 0,50 Jahre; Max = 40,00 Jahre).

### Zufriedenheit mit dem SOLAR-Programm

Entsprechend dem ZUF-8-Gesamt-Score, bei dem maximal 32 Punkte erreicht werden können, gaben die Teilnehmenden der Präsenzgruppe (Mittelwert [M] = 29,7 Punkte, SD ± 2,72 Punkte) und der Onlinegruppe (M = 24,45 Punkte, SD ± 4,27 Punkte) im Mittel eine hohe Zufriedenheit an (Tab. 2). Dabei zeigte sich deskriptiv, dass die Teilnehmenden der Präsenzgruppe im Vergleich zur Onlinegruppe im Durchschnitt über alle Zufriedenheitsaspekte geringfügig zufriedener mit dem Programm waren.

**Table 2:** 

	SOLAR-Präsenzgruppe (*n* = 13)				SOLAR Onlinegruppe (*n* = 11)			
	M	± SD	Min	Max	M	± SD	Min	Max
*Gesamtzufriedenheit*	*29,66*	± *2,72*	*23*	*32*	*24,45*	± *4,27*	*16*	*30*
*Einzelne Zufriedenheitsaspekte*								
1. Wie würden Sie die Qualität des Programms, das Sie erhalten haben, beurteilen?^a^	3,54	± 0,52	3	4	3,18	± 0,60	2	4
2. Haben Sie die Art des Programms erhalten, die Sie wollten?^b^	3,62	± 0,65	2	4	2,91	± 0,54	2	4
3. In welchem Maße hat unser Programm Ihren Bedürfnissen entsprochen?^c^	3,31	± 0,63	2	4	2,73	± 0,65	1	3
4. Würden Sie einem Freund/einer Freundin unser Programm empfehlen, wenn er/sie eine ähnliche Hilfe benötigen würde?^d^	3,77	± 0,44	3	4	3,27	± 0,79	2	4
5. Wie zufrieden sind Sie mit dem Ausmaß der Hilfe, die Sie hier erhalten haben?^e^	3,85	± 0,38	3	4	3,09	± 0,54	2	4
6. Hat das Programm, das Sie hier erhielten, Ihnen dabei geholfen, angemessener mit Ihren Problemen umzugehen?^f^	3,92	± 0,27	3	4	3,00	± 0,78	2	4
7. Wie zufrieden sind Sie mit dem Programm, das Sie erhalten haben, im Großen und Ganzen?^g^	3,92	± 0,27	3	4	3,09	± 0,70	2	4
8. Würden Sie wieder an unserem SOLAR-Programm teilnehmen, wenn Sie Hilfe bräuchten?^h^	3,77	± 0,59	2	4	3,18	± 0,75	2	4

*SOLAR* „Skills for Life Adjustment and Resilience Program“

^a^*1* Schlecht, *2* weniger gut, *3* gut, *4* ausgezeichnet

^b^*1* Eindeutig nicht, *2* eigentlich nicht, *3* i. Allg. ja, *4* eindeutig ja

^c^*1* Sie hat meinen Bedürfnissen nicht entsprochen. *2* Sie hat nur wenigen meiner Bedürfnissen entsprochen. *3* Sie hat den meisten meiner Bedürfnisse entsprochen. *4* Sie hat fast allen meinen Bedürfnissen entsprochen

^d^*1* Eindeutig nicht, *2* eigentlich nicht, *3* i. Allg. ja, *4* eindeutig ja

^e^*1* Ziemlich unzufrieden, *2* leicht unzufrieden, *3* weitgehend zufrieden, *4* sehr zufrieden

^f^*1* Nein, es hat mir die Dinge schwerer gemacht. *2* Nein, es half eigentlich nicht. *3* Ja, es half etwas. *4* Ja, es half eine ganze Menge

^g^*1* Ziemlich unzufrieden, *2* leicht unzufrieden, *3* weitgehend zufrieden, *4* sehr zufrieden

^h^*1* Eindeutig nicht, *2* eigentlich nicht, *3* i. Allg. ja, *4* eindeutig ja

Die Teilnehmenden der Präsenzgruppe waren im Mittel „sehr zufrieden“ (M = 3,92, SD ± 0,27). Sie bejahten eindeutig die Aussage, dass das Programm ihnen dabei geholfen habe, angemessener mit ihren Problemen umzugehen (M = 3,92, SD ± 0,27). Im Vergleich zu den anderen Zufriedenheitsaspekten wurde der niedrigste Wert bei der Frage erreicht, inwiefern das Programm den Bedürfnissen der Teilnehmenden entsprochen habe (M = 3,31, SD ± 0,63). Hierbei wurde im Mittel angegeben, dass „die meisten Bedürfnisse“ erfüllt worden waren.

Die Teilnehmenden der Onlinegruppe gaben im Mittel die höchsten Werte bei den Fragen zur Qualität des Programms sowie zur Weiterempfehlung an. Die Qualität des Programms wurde im Mittel als „gut“ bewertet (M = 3,18, SD ± 0,60). Die Teilnehmenden gaben an, das sie das Programm weiterempfehlen würden (M = 3,27, SD ± 0,79). Übereinstimmend mit den Ergebnissen der Präsenzgruppe fanden sich in der Onlinegruppe die niedrigsten Werte bei der Frage zur Übereinstimmung des Programms mit ihren individuellen Bedürfnissen (M = 2,73, SD ± 0,65); hier gaben die Teilnehmenden im Mittel „den meisten Bedürfnissen entsprochen“ an.

## Diskussion

Anhaltend subklinisch belastete Menschen stellen eine Gruppe dar, die oftmals keine psychosoziale Unterstützung erhält. Das SOLAR-Programm wurde entwickelt, um diese Praxislücke zu schließen. Im Rahmen der vorgestellten Studie wurde das SOLAR-Programm erstmals im Präsenz- und im Videokonferenzformat in Deutschland bei anhaltend subklinisch belasteten Teilnehmenden mit traumatischen Erfahrungen erprobt, und die Teilnehmenden wurden gebeten, Auskunft über ihre Zufriedenheit mit dem Programm zu geben.

Die Teilnehmenden gaben bei allen 8 gemessenen Zufriedenheitsaspekten an, mit dem SOLAR-Gruppenprogramm sowohl im Präsenz- als auch im Onlineformat weitgehend bis sehr zufrieden zu sein. Die Qualität des Programms wurde als gut bewertet, und die Teilnehmenden gaben an, dass das Training ihnen geholfen habe, angemessener mit ihren Problemen umzugehen. Übereinstimmend mit diesen Ergebnissen berichteten die Teilnehmenden in der Feedbackrunde der letzten Programmsitzung, dass sie mit den Programminhalten zufrieden gewesen seien. Die Präferenzen für bestimmte inhaltliche Bereiche unterschieden sich jedoch zwischen den Teilnehmenden. So berichteten einige Teilnehmenden, dass sie sich bereits gesund ernähren würden und dieser Themenbereich daher für sie weniger relevant sei; sie hätten lieber mehr Zeit für andere Themenbereiche genutzt, wie z. B. den Umgang mit Grübeln. Andere Teilnehmende fanden wiederum den Themenbereich der gesunden Ernährung besonders hilfreich. Diese Ergebnisse weisen darauf hin, dass je nach Interessenlage der Gruppe Schwerpunkte gesetzt werden könnten. Die Übungen zum Umgang mit Sorgen und Grübeln trafen bei allen Teilnehmenden auf großen Zuspruch. Die Schreibaufgabe zum belastenden Ereignis wurde ebenfalls von allen Teilnehmenden als besonders bedeutsam eingeschätzt.

### Empfehlungen zur Durchführung des SOLAR-Programms

Auf der Grundlage ihrer gemachten Erfahrungen haben die TrainerInnen des SOLAR-Programms dieser Studie gemeinsam Empfehlungen für die Durchführung entwickelt; diese sind am Ende dieses Beitrags im Abschn. „Fazit für die Praxis“ in Kurzform dargestellt und werden im Folgenden eingehender erläutert. Im Rahmen der Gewinnung von Gruppenteilnehmenden erwies es sich als hilfreich, den Begriff eines „Programms“ anstelle des Begriffs der „Psychotherapie“ zu verwenden, da sich das SOLAR-Programm nicht an Personen mit psychischer Erkrankung (mit Ausnahme der Anpassungsstörung) richtet und der Begriff des „Programms“ von den meisten Teilnehmenden als weniger stigmatisierend erlebt wurde. Um eine hohe Durchführungsqualität zu gewährleisten und die psychische Belastung der TrainerInnen zu reduzieren, sollten diese von einer psychotherapeutisch erfahrenen Fachkraft vorab geschult und regelmäßig supervidiert werden. Dies erscheint zum einen bedeutsam, um zu gewährleisten, dass die Programmkonzepte richtig verstanden und an die Teilnehmenden vermittelt wurden; zum anderen, um die TrainerInnen in schwierigen Situationen zu unterstützen. Die Durchführung des Programms in Teams aus 2 TrainerInnen bietet zudem die Möglichkeit der gegenseitigen Unterstützung. Eine frühzeitige Planung der Gruppentermine erschien von besonderer Bedeutung, da die meisten Teilnehmenden berufstätig waren. Vor diesem Hintergrund sollten die Termine am späten Nachmittag oder am Abend stattfinden. Aus der Perspektive der TrainerInnen war das SOLAR-Gruppenprogramm durch die Schulung und die manualisierte Vorgehensweise auch für weniger erfahrene TrainerInnen gut durchführbar. Im Gruppenformat mit 5 bis 9 Teilnehmenden erwies sich eine Gruppendauer der Sitzungen von 100 min für die erste Sitzung und 90 min für die weiteren Sitzungen als angemessen, um ausreichend Zeit für die zu vermittelnden Inhalte und Übungen zu haben und den Austausch zwischen den Teilnehmenden zu ermöglichen.

Setzt sich die Gruppe aus Teilnehmenden mit unterschiedlichen Traumata zusammen, erwies es sich in der ersten Gruppensitzung als vorteilhaft, diese nicht dazu zu ermutigen, über die Art des traumatischen Ereignisses zu berichten. Dies kann einer möglichen Stigmatisierung bei bestimmten Formen von Traumata (z. B. sexuelle Gewalt vs. Autounfall) und Vergleichen zwischen verschiedenen Schweregraden von Traumatisierungen vorbeugen.

Dagegen erscheint es wichtig, die Teilnehmenden wiederholt dazu zu ermutigen, die Schreibaufgabe zum belastenden Ereignis durchzuführen, um eine emotionale Verarbeitung zu ermöglichen. Hierbei sollte ausreichend Zeit für die Einführung der Aufgabe zur Verfügung stehen, damit die Teilnehmenden nachvollziehen können, warum es hilfreich sein kann, sich der belastenden Situation auszusetzen. Den Teilnehmenden wurde das Prinzip des Angstabfalls durch Habituation erläutert, sowie die Rolle von Vermeidungsverhalten für die Aufrechterhaltung der Angst. Die meisten Teilnehmenden konnten das Rational hinter der Schreibaufgabe gut nachvollziehen. Es war jedoch bedeutsam, Befürchtungen gegenüber der Aufgabe als verständlich und nachvollziehbar zu validieren und die Teilnehmenden wiederholt zu ermutigen, die Aufgabe durchzuführen. Auch sollten mögliche Vermeidungsstrategien vorab angesprochen werden, sowie Strategien, die die Durchführung erleichtern.

Neben der Durchführung der Schreibaufgabe erschien aus der Sicht der TrainerInnen die positive Bestärkung der Teilnehmenden, die wöchentlichen Aufgaben durchzuführen, als zentral. Für einige Teilnehmende war es zunächst ungewohnt, selbst aktiv zu werden und Verhaltensänderungen in ihren Alltag zu integrieren. Umso wichtiger war daher die Nachbesprechung der Aufgaben in der nächsten Sitzung. So konnte reflektiert werden, was bei den Probanden bereits gut oder noch nicht so gut funktioniert hatte. Zudem konnte besser nachvollzogen werden, aus welchen Gründen bestimmte Verhaltensweisen noch nicht umgesetzt werden konnten, und welche Strategien helfen könnten, die neuen positiven Gewohnheiten im Alltag umzusetzen. Um den Transfer in den Alltag zu unterstützen, erwies es sich als hilfreich, in jeder Sitzung eine Übung gemeinsam mit den Teilnehmenden praktisch zu erproben.

### Anwendung des SOLAR-Programms im Onlineformat während der COVID-19-Pandemie

Vor dem Hintergrund der beginnenden COVID-19-Pandemie im Frühjahr 2020 wurde das SOLAR-Programm bei 2 Gruppen von Beginn an onlinegestützt mithilfe einer Videokonferenz durchgeführt. Die Ergebnisse zur Zufriedenheit der Teilnehmenden weisen darauf hin, dass die Teilnehmenden auch mit dem Onlineprogramm zufrieden waren, wenngleich die Zufriedenheit im Vergleich zum Präsenzformat geringfügig niedriger ausfiel. Die etwas geringere Zufriedenheit könnte darauf zurückzuführen sein, dass das Programm ursprünglich als Präsenzprogramm angekündigt worden war und die Teilnehmenden eine entsprechende Erwartung entwickelt hatten. Mehrere der Teilnehmenden hatten zuvor wenig oder keine Erfahrung mit onlinegestützten Gesundheitsmaßnahmen, sodass die onlinegestützte Durchführung zu Beginn bei einem Teil der Teilnehmenden Gewöhnung benötigte. Diese Teilnehmenden berichteten jedoch, dass sie sich rasch an den neuen Kommunikationsweg gewöhnt hätten, und dass die Inhalte und neu zu erlernenden Fertigkeiten auch online gewinnbringend vermittelt werden konnten. Keiner der Teilnehmenden sagte die Teilnahme am Programm aufgrund der Umstellung auf das onlinebasierte Format ab. Ein Teilnehmer konnte jedoch aufgrund von technischen Schwierigkeiten ab der 3. Sitzung nicht mehr an dem Programm teilnehmen. Es ist davon auszugehen, dass die Akzeptanz eines solchen Formates aufgrund der im letzten Jahr stark zugenommenen Vertrautheit mit onlinebasierten Maßnahmen deutlich zugenommen hat. Die Teilnehmenden betonten als Vorteil des Onlineprogramms, dass dieses durch den Wegfall von Fahrtzeiten einfacher mit ihrer beruflichen Tätigkeit vereinbar gewesen sei. Mehrere TeilnehmerInnen betonten, dass die onlinegestützte Durchführung gerade zum Zeitpunkt der beginnenden COVID-19-Pandemie hilfreich gewesen sei, mit den besonderen Belastungen dieser Ausnahmesituation umzugehen.

Von den TrainerInnen wurde das onlinebasierte Programmformat ebenfalls als gut durchführbar eingeschätzt. Voraussetzung war jedoch, dass alle Teilnehmenden über einen internetfähigen Computer oder ein Smartphone sowie über ein Mikrofon und eine Kamera verfügten. Letztere sollte während der gesamten Sitzung angeschaltet bleiben, um die Gesprächsdynamik innerhalb der Gruppe aufrechtzuerhalten und den Austausch zwischen den Teilnehmern zu gewährleisten. Es erwies sich als hilfreich, frühzeitig über nötige technische Vorbereitungen zu informieren (z. B. Prüfen der Funktionsfähigkeit von Kamera und Mikrofon) und diese bei Bedarf gemeinsam mit den Teilnehmenden vorab zu testen und ggf. vorhandene technische Probleme zu lösen, um technischen Schwierigkeiten in der ersten Sitzung vorzubeugen. Auch wenn die Sitzungen sensible Inhalte betrafen, wurde das onlinebasierte Setting nicht als Barriere empfunden, über diese Inhalte zu sprechen. Besonders zu beachten waren die Einhaltung der geltenden Datenschutzregelungen und das Einverständnis aller Teilnehmenden mit diesen Regelungen, etwa die Unterlassung der Aufzeichnung der Sitzungen. Es sollte zudem vor Beginn der ersten Onlinesitzung besprochen werden, dass die Teilnehmenden und die TrainerIn für die Dauer der Sitzung ungestört sein sollten.

Insgesamt zeigen die berichteten ersten Praxiserfahrungen, dass das SOLAR-Programm gut durchführbar war und die Teilnehmenden mit dem Programm weitgehend bis sehr zufrieden waren. Das SOLAR-Programm könnte im Rahmen eines gestuften Versorgungsansatzes in Ergänzung zu psychotherapeutischen Maßnahmen für anhaltend subklinisch belastete Menschen nach schweren Belastungen in psychosozialen Einrichtungen wie z. B. Beratungsstellen angeboten werden. Aber auch im klinischen Kontext, z. B. in tagesklinischen Einrichtungen, könnte es als Gruppenprogramm in Ergänzung zu einzeltherapeutischen Angeboten hilfreich sein.

Ein großes Potenzial könnte die onlinegestützte Anwendung des Programms bieten. So könnte das Programm für anhaltend subklinisch belastete Menschen eingesetzt werden, die während der COVID-19-Pandemie besonderen Belastungen ausgesetzt waren. Auf diese Weise könnten Menschen trotz Kontaktbeschränkungen Unterstützung erhalten. Dies könnte gerade auch für weniger mobile Menschen von Bedeutung sein, und auch für Menschen in ländlichen Regionen mit schwachen Versorgungsstrukturen. Hierzu ist es jedoch zunächst notwendig, die Effektivität des SOLAR-Programms im Rahmen größerer randomisierter kontrollierter Studien zu überprüfen.

## Schlussfolgerung

Insgesamt weisen die Ergebnisse dieser Studie auf die Durchführbarkeit und Akzeptanz des SOLAR-Programms hin. Das Programm könnte im Rahmen eines gestuften Versorgungsansatzes in Ergänzung für anhaltend subklinisch belastete Menschen nach Katastrophen und anderen schweren Belastungen eingesetzt werden, sofern es sich als wirksam erweisen sollte.

## Fazit für die Praxis

Empfehlungen für TrainerInnen zur Durchführung des SOLAR-Programms:

- Nehmen Sie an einer 2‑tätigen SOLAR-Schulung durch einen/eine „SOLAR Train the TrainerIn“ teil, um die Anwendung des Programms zu erlernen. Schulungsmöglichkeiten können bei der Erstautorin erfragt werden.
- Verwenden Sie den Begriff „Programm“ anstelle von „Psychotherapie“, wenn Sie Interessenten über das SOLAR-Programm informieren, um Menschen mit subklinischer Belastung anzusprechen.
- Während der Durchführung des Programms sollten Sie regelmäßig von einer erfahrenen Gesundheitsfachkraft mit Erfahrungen in der Behandlung von Traumafolgestörungen supervidiert werden.
- Ein/eine KotrainerIn sollte die/den TrainerIn bei der Durchführung des Gruppenprogramms unterstützen.
- Um Berufstätigen die Teilnahme zu ermöglichen, sollte das Programm in den Nachmittags- oder Abendstunden angeboten werden.
- Das Berichten über die Art des traumatischen Ereignisses sollte in der ersten Gruppensitzung bei Gruppen mit unterschiedlichen Traumatisierungstypen nicht angeboten werden, um Stigmatisierung und Vergleiche zwischen verschiedenen Schweregraden zu vermeiden.
- Ermutigen und unterstützen Sie die Teilnehmenden bei der Aufgabe, das belastende Ereignis aufzuschreiben, da diese Aufgabe herausfordernd sein kann.
- Bestärken Sie die Teilnehmenden in der Durchführung der wöchentlichen Aufgaben zwischen den Sitzungen und betonen Sie deren Bedeutsamkeit für die Wirksamkeit des Programms.
- Führen Sie in jeder Sitzung gemeinsame Übungen mit den Teilnehmenden durch, um den Transfer in die Praxis zu unterstützen.
